# Adenomyosis-Modern Techniques for Ultrasound and Histo-Pathological Diagnosis of the Endo-Myometrial Junction Zone Changes

**DOI:** 10.3390/jcm14248744

**Published:** 2025-12-10

**Authors:** Elena Iuliana Anamaria Berbecaru, George-Lucian Zorilă, Anca-Maria Istrate-Ofiţeru, Gabriela-Camelia Roșu, Elvira Brătilă, Daniel Pirici, Cristina Jana Busuioc, Laurențiu Mogoantă, Răzvan Grigoraș Căpitănescu, Dominic-Gabriel Iliescu, Marian Valentin Zorilă

**Affiliations:** 1 Doctoral School, University of Medicine and Pharmacy of Craiova, 200349 Craiova, Romania; elena.berbecaru@umfcv.ro; 2 Department of Obstetrics and Gynecology, University of Medicine and Pharmacy of Craiova, 200349 Craiova, Romania; lucian.zorila@umfcv.ro; 3 Department of Histology, Faculty of Medicine, University of Medicine and Pharmacy of Craiova, 200349 Craiova, Romania; camelia.rosu@umfcv.ro (G.-C.R.); daniel.pirici@umfcv.ro (D.P.); cristina.busuioc@umfcv.ro (C.J.B.); laurentiu.mogoanta@umfcv.ro (L.M.); 4 Research Center for Microscopic Morphology and Immunology, University of Medicine and Pharmacy of Craiova, 200349 Craiova, Romania; 5 Department of Obstetrics and Gynecology, Carol Davila University of Medicine and Pharmacy, 050474 Bucharest, Romania; elvira.bratila@umfcd.ro; 6 Department of Forensic Medicine, University of Medicine and Pharmacy of Craiova, 200349 Craiova, Romania; valentin.zorila@umfcv.ro

**Keywords:** adenomyosis, infertility, HyCoSy

## Abstract

**Background/Objectives**: Adenomyosis (A) is a benign but invasive uterine condition frequently associated with structural changes in the uterine wall that may contribute to infertility. **Methods**: This is a retrospective study involving 140 patients: 100 diagnosed with primary infertility (PI) or secondary infertility (SI) and 40 in the control group. All patients were assessed using transvaginal two-dimensional, three-dimensional, and hysterosalpingo-contrast sonography (HyCoSy), performed in the early proliferative phase. Evaluated parameters included uterine dimensions, endometrial thickness, and characteristics of the junctional zone (JZ). Criteria such as JZmax > 5 mm or JZmax − JZmin > 5 mm, alongside other findings, supported the diagnosis of adenomyosis. **Results**: Patients with PI showed larger uterine longitudinal diameters, while SI patients had thicker JZ measurements. PI patients were significantly younger. Histopathological examination confirmed the presence of endometrial glands and periglandular stroma disrupting myometrial architecture, forming chronic lesions potentially linked to infertility. **Conclusions**: HyCoSy revealed variable depths of myometrial invasion by A, with some cases extending near the serosa. The chronic lesions found in histopathological examination were potentially linked to infertility.

## 1. Introduction

Adenomyosis (A) is a benign uterine disorder defined by the presence of ectopic endometrial glands and stroma within the myometrium. The endometrial glands extend at least 2.5 mm beneath the basal endometrium, frequently leading to uterine enlargement [1]. Traditionally considered a condition of multiparous women aged 40–50 years, A is increasingly recognized in younger patients, with 5–25% of cases diagnosed before the age of 39 [2]. The disease frequently coexists with uterine fibroids (35–55%) and endometriosis (65–70%) [3]. Reported risk factors include multiparity, age over 40 years, and previous uterine surgery such as cesarean section [4].

Clinically, approximately two-thirds of affected women present with symptoms including dysmenorrhea, heavy menstrual bleeding, chronic pelvic pain, and infertility [5]. Beyond its symptomatic burden, A is increasingly recognized as an important factor in reproductive medicine. Evidence suggests detrimental effects on both spontaneous conception and assisted reproductive technology (ART) outcomes [6]. Several systematic reviews and meta-analyses confirm the negative impact of A on fertility, showing reduced clinical pregnancy rates, higher miscarriage rates (31% vs. 14.1% in controls), and impaired live birth outcomes [7,8,9]. More recently, large cohort analyses have also demonstrated an association between A and adverse perinatal outcomes [10].

The underlying mechanisms by which A contributes to infertility remain incompletely understood, partly due to the frequent coexistence of endometriosis. Hypothesized pathways include disruption of myometrial architecture, abnormal uterine contractility, impaired uterine peristalsis, elevated intrauterine pressure, altered steroid hormone signaling, chronic inflammation, oxidative stress, and downregulation of implantation-related molecular markers [11]. Importantly, alterations of the junctional zone (JZ)—the inner myometrium critical for uterine peristalsis and implantation—are believed to play a central role [12].

The JZ can be accurately assessed in the coronal plane using three-dimensional transvaginal sonography (3D-TVUS). Morphological abnormalities of the JZ observed by 3D ultrasound have been correlated with A and provide high diagnostic accuracy [12]. According to the Morphological Uterus Sonographic Assessment (MUSA) group, the myometrium is divided into three layers: the JZ (inner myometrium), the middle myometrium (extending to the arcuate vascular plexus), and the outer myometrium adjacent to the serosa [12]. The JZ was first described as a hypoechoic halo surrounding the endometrium [13,14]. While two-dimensional transvaginal ultrasonography (2D-TVUS) provides limited evaluation, 3D-TVUS allows complete multiplanar assessment and superior visualization, especially when combined with volume contrast imaging (VCI) [15,16]. JZ visibility may vary according to endometrial thickness, parity, or fibroid presence [17], although some studies found no correlation with demographic or hormonal factors [18].

Despite these advances, adequate JZ visualization remains technically challenging. In a study by Rasmussen et al., even in expert hands, JZ was visualized in only 44% of sagittal and 68% of coronal views [19,20]. Sonographic features of A include direct signs—such as hyperechoic sub endometrial lines, nodular projections, and myometrial cysts—and indirect signs including myometrial heterogeneity [12,15,21,22]. However, similar features may also occur in other uterine pathologies, including endometrial carcinoma with myometrial invasion [23]. Recent consensus statements emphasize that diagnosis should not rely solely on JZ thickness, but rather on the integration of multiple sonographic features [15,24,25,26].

Although hysterosalpingo-contrast sonography (HyCoSy) is primarily performed for the evaluation of tubal patency in women with infertility, recent evidence suggests it may also provide valuable information regarding uterine morphology. In transvaginal 4D-HyCoSy, A has been associated with a significantly higher incidence of contrast agent reflux, reflecting potential JZ disruption and altered myometrial architecture [27]. Moreover, HyCoSy has been shown to be a safe, cost-effective, and practical diagnostic tool, with some studies reporting spontaneous pregnancies following the procedure in women with unexplained infertility [28]. Recent reviews further emphasize its clinical relevance, highlighting both its diagnostic potential and its limitations, such as operator dependency and variability in technique [29]. When integrated into the infertility work-up, HyCoSy therefore offers a broader perspective, simultaneously addressing tubal and uterine factors, and complementing 2D/3D-TVUS and MRI [22,30].

Histologically, A is characterized by the presence of endometrial glands and stroma infiltrating ≥2–2.5 mm into the myometrium [31,32]. However, diagnostic thresholds remain debated, as the transition between inner and outer myometrium is gradual and superficial invasion may be physiological [33,34]. Structural abnormalities of the JZ include smooth muscle cell (SMC) hyperplasia, nuclear enlargement, altered cell density, and the presence of myofibroblasts indicative of chronic tissue microtrauma [35,36,37]. Immunohistochemistry reveals altered α-smooth muscle actin (α-SMA) expression, increased collagen deposition, and extracellular matrix remodeling, findings consistent with chronic injury and repair [36,38,39].

Histopathological confirmation remains the gold standard for diagnosing A, typically defined by ectopic endometrial glands and stroma located at least 2–2.5 mm beyond the endometrial–myometrial junction [31,32,34]. Microscopic studies have demonstrated smooth muscle cell disorganization, hypertrophy, and extracellular matrix remodeling in the JZ, as well as increased vascular density at this level [35,36,37]. Immunohistochemical analyses further support these findings, with increased α-smooth muscle actin (α-SMA) expression indicating myofibroblast proliferation and CK7 staining highlighting glandular invasion [38,39]. Such correlations between imaging and histology may help clarify the mechanisms by which A impairs uterine function and fertility.

Despite advances in histology and imaging, the lack of standardized diagnostic criteria and classification systems leads to variability in prevalence estimates and clinical interpretation [5,34,40]. A major limitation remains the heterogeneity of outcome reporting across studies, underscoring the urgent need for harmonized diagnostic frameworks [41]. Modern approaches propose phenotype-based classifications linking imaging and histology with clinical outcomes [34,40]. Furthermore, emerging perspectives suggest that advanced ultrasound techniques, MRI protocols, and artificial intelligence may enhance reproducibility and diagnostic accuracy in A [42].

The aim of this study is to evaluate the morphological changes in the endo-myometrial JZ using 3D ultrasound, and to assess endometrial invasion through contrast-enhanced HyCoSy in infertile patients who wish to preserve their fertility. The study further aims to correlate the 3D ultrasound features of myometrial invasion with histopathological characteristics and the depth of glandular invasion into the myometrial structure.

## 2. Materials and Methods

This retrospective study included 140 women who agreed to participate and were examined between 2018 and 2024 at the Clinical County Hospital of Craiova. The study group comprised 100 patients (71.4%) diagnosed with A based on ultrasound criteria. Within this group, some presented with primary infertility (PI) and others with secondary infertility (SI).

The control group consisted of forty patients (28.6%), of which twenty (14.28%) were young women without uterine pathology who had died in road traffic accidents and whose families provided informed consent for histological analysis, and the other twenty were patients without gynecological pathology who consented to undergo complete transvaginal ultrasound (2D-TVUS and 3D-TVUS) and contrast-enhanced HyCoSy for comparative evaluation. To improve clarity, we specify that the control group included two distinct subgroups with different purposes: (B1) post-mortem controls used exclusively for histopathological comparison, and (B2) living volunteers used exclusively for ultrasound and HyCoSy imaging reference values. These two subgroups were not merged for any direct statistical comparison.

Written informed consent was obtained from all participants or their legal representatives. The study was conducted in accordance with the Declaration of Helsinki and was approved by the Ethics and Scientific Deontology Committee of the University of Medicine and Pharmacy of Craiova (approval no. 88/13 September 2018).

All women with PI or SI and the twenty ultrasound-evaluated controls underwent clinical and sonographic assessment. Only the living volunteers (*n* = 20) served as imaging controls for ultrasound-based parameters, including uterine dimensions, JZ thickness, myometrial features, and tubal patency assessed by HyCoSy. Diagnosis of A and infertility was based on clinical and ultrasound assessment, transabdominal and transvaginal (2D grayscale and color Doppler) performed with a Voluson E10 ultrasound system (GE Healthcare), equipped with 3–5 MHz transabdominal and 3–9 MHz transvaginal probes. Evaluation followed established sonographic criteria focusing on characteristics of the end myometrial JZ. All ultrasound features were described according to the standardized terminology and definitions of the MUSA consensus, including junctional zone thickness, subendometrial echogenic lines, hypoechoic striations, myometrial cysts, and JZ regularity.

Ultrasound findings were evaluated in accordance with the 2019 adenomyosis classification and reporting recommendations proposed by Van den Bosch et al. [12], which provide a standardized framework for describing lesion type, location, extent, and junctional zone characteristics. All JZ measurements were obtained using standardized 3D multiplanar reconstruction, and scans were performed by two senior operators with over 10 years of experience in gynecologic ultrasound. Ambiguous cases were reviewed in consensus to ensure consistency and reduce inter-observer variability.

Surgical treatment (total hysterectomy) was performed in 40 patients (40%) who no longer desired fertility preservation. Uterine tissue samples from these patients were fixed in 10% formalin and processed via paraffin embedding for histological analysis.

All patients attempting conception underwent additional evaluation of the uterine cavity, fallopian tubes, and tubal patency by HyCoSy using transcervical infusion of SonoVue^®^ contrast agent (8 mL/vial, Bracco, Milan, Italy) through a Foley catheter. The investigation was performed during the early proliferative phase. Prior to contrast administration, 2D- and 3D-TVUS scans were performed with multiplanar reconstructions to evaluate the uterine size, the endometrial thickness, the minimum and maximum JZ thickness (JZmin, JZmax), the asymmetry of myometrial walls, and the presence of myometrial cysts, hyperechoic areas, and hypoechoic striations.

The presence of A was considered when JZmax > 5 mm or JZmax – JZmin > 5 mm in association with other ultrasound features These findings were correlated, when possible, with the etiology of infertility.

Each patient completed a form indicating age and infertility type (PI—group “A1” or SI—group “A2”). All collected data were centralized using Microsoft Excel, and average measurements were determined as follows: age, uterine dimensions, minimum and maximum JZ thickness, endometrial thickness, prevalence (%) of cystic lesions, presence of hyperechoic areas, hypoechoic striations, CD34+ vascular density, and smooth muscle cell density in both cases in group A2 and control cases in group B1. Statistical analysis was performed using GraphPad Prism 10—one-way ANOVA to compare mean values across the four study groups (A1, A2, B1, B2), followed by Tukey HSD post hoc testing for pairwise comparisons. Welch’s t-test (Two-Sample Assuming Unequal Variances) was applied for specific two-group comparisons where appropriate. Histological parameters were compared between adenomyosis cases and the post-mortem control subgroup, whereas ultrasound and HyCoSy parameters were compared only with the living control subgroup. No combined statistical analysis of the two control subgroups was performed. The figures display mean values and standard deviations (SDs). Regarding vascular and cellular densities, an average of 4 images were captured in JZ with a 20× objective from each specimen, using constant manual exposure and illumination settings. All elements (cells, blood vessels) were counted manually using the “manual tag” function in Image ProPlus 6.0, the average was calculated for each slide, then for each subgroup analyzed. The final percentage averages were graphically represented using Microsoft Excel, and for statistical analysis, we used the same comparative statistical tests. All histological and immunohistochemical assessments, including SMC and vascular density quantification, were performed by a senior histopathologist blinded to group allocation and clinical data. Cell counting was conducted on standardized ×200 fields using identical illumination and exposure settings, and values were recorded before unblinding for statistical analysis.

Tissue samples were analyzed in the Department of Pathology, Clinical County Emergency Hospital of Craiova, and in the Research Center for Microscopic Morphology and Immunology, University of Medicine and Pharmacy of Craiova. Tissue specimens were fixed in 4% neutral buffered formaldehyde, embedded in paraffin, and sectioned at 4 μm thickness using a microtome. A diagnosis was established using both histopathological and immunohistochemical methods. Post-mortem control specimens were used only to characterize the normal histological structure of the JZ and myometrium, serving as a reference for histopathological comparisons with A cases. Post-mortem control specimens were included because healthy uterine tissue from young women without gynecological pathology is rarely available from surgical cases. These samples were used exclusively for histopathological reference, whereas all ultrasound and HyCoSy analyses were performed only in living participants.

The immunohistochemical procedure was carried out following a standard protocol, adjusted according to the specific antibodies used. Antigen retrieval was performed by microwave heating in citrate buffer (pH 6.0), in accordance with the manufacturers’ instructions. Subsequently, the slides were incubated for 30 min in 3% hydrogen peroxide to block endogenous peroxidase activity, followed by incubation in a 3% skim milk saline solution to prevent nonspecific antibody binding. Primary antibodies (Table 1) diluted according to the manufacturers’ recommendations (Table 1; Dako, Glostrup, Denmark; Abcam, Cambridge, UK), were applied overnight at 4 °C followed by HRP-conjugated secondary antibodies (Nikirei-Bioscience, Tokyo, Japan). Detection was achieved with 3,3′-diaminobenzidine (DAB; Nikirei-Bioscience), and sections were counterstained with Mayer’s hematoxylin, dehydrated, cleared in xylene, and mounted with Canadian balsam for microscopic examination.

## 3. Results

We analyzed a group of 140 women from which 100 had A (Group A) and infertility and the other 40 represented the control group (Group B).

The patients wishing to preserve the fertility were allocated in Group A1 (*n* = 60), the ones that choosing total hysterectomy were allocated in Group A2 (*n* = 40), and the control group was Group B, which comprised 40 controls with histologically normal endo-myometrium (20 post-mortem specimens without uterine pathology—group B1; or comprised 20 healthy volunteers evaluated by TVUS/3D-TVUS/HyCoSy—group B2).

All patients with PI expressed a desire to preserve their uterus and fertility, whereas all patients with SI included in the study opted for total hysterectomy in order to minimize their symptoms.

### 3.1. Age Distribution of the Study Population

**Figure 1:**
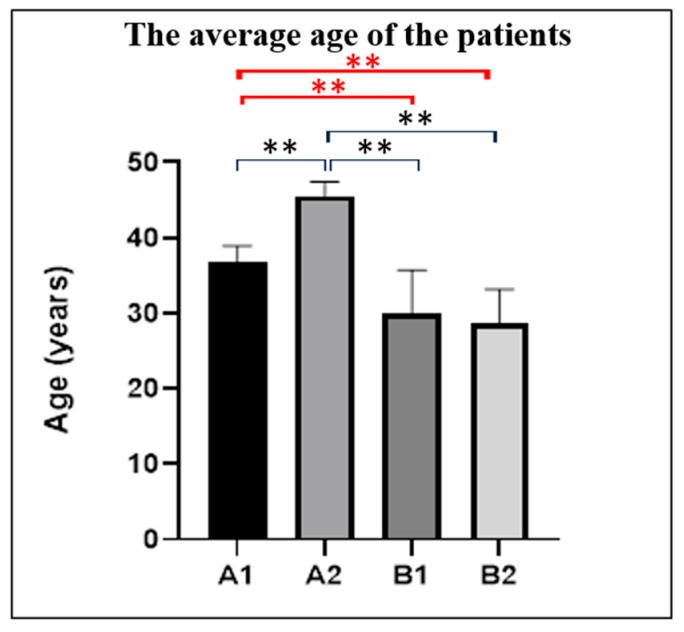
Mean age of patients in each study group. Data are presented as individual values for each subject, overlaid with group means ± SEM. A one-way ANOVA revealed a significant effect of group (F(3,96) = 167.9, *p* < 0.0001, R^2^ = 0.7874). Tukey’s post hoc test showed that A2 exhibited significantly higher values than all other groups (*p* < 0.0001), and A1 was significantly higher than both B1 and B2 (*p* < 0.0001). No significant difference was observed between B1 and B2 (*p* = 0.5836). ** in red indicates *p* < 0.0001; ns, not significant. A1: The group of patients with adenomyosis and primary infertility. A2: The group of patients with adenomyosis and secondary infertility. B1: The control group that included women who died in road traffic accidents, from whom healthy tissues were used for histopathological analysis. B2: The control group that included healthy patients whose imaging findings were used for comparative studies.

Mean age in Group “A” was 40.28 ± 4.47 years, ranging from 34 to 49 years vs. a mean age of 29.3 ± 5.16 years in Group “B”, ranging from 21 to 39 years. In the A1 group, age ranged from 34 to 41 years, with a mean value of 36.8 years (±2.12 years); in the A2 group, age ranged from 42 to 49 years, with a mean value of 45.5 years (±1.88 years); in the B1 group, age ranged from 21 to 39 years, with a mean value of 29.95 years (±5.74 years); and in the B2 group, age ranged from 21 to 35 years, with a mean value of 28.65 years (±4.55 years). A one-way ANOVA revealed a significant effect of group on the measured values (F(3,96) = 167.9, *p* < 0.0001, R^2^ = 0.7874). Tukey’s post hoc test showed that group A2 exhibited significantly higher values compared with all other groups (*p* < 0.0001), while group A1 also differed significantly from both B1 and B2 (*p* < 0.0001). In contrast, no significant difference was observed between B1 and B2 (*p* = 0.5836) (Figure 1). Overall, age distribution differed significantly between groups. Patients with adenomyosis (Group A) were consistently older than healthy controls (Group B). Within Group A, women with secondary infertility (A2) were the oldest subgroup, with significantly higher ages compared with all other groups, including those with primary infertility (A1). Meanwhile, both A1 and A2 were significantly older than the control subgroups B1 and B2, between which no significant age differences were observed. These findings indicate a clear age-related pattern, with adenomyosis being associated with a substantially higher patient age, particularly in cases of secondary infertility.

### 3.2. Clinical Data of the Pathological Group

When analyzing the symptoms of the patients in Group A, we observed that 78% of the A1 subgroup presented with dysmenorrhea (Dy), 65% presented with menorrhagia (Men), 22% reported metrorrhagia (Met), and only 10% mentioned elevated blood pressure values above 140/90 mmHg (Hy). In the A2 subgroup, 100% of the patients presented with Dy and Men, 43% presented with Met, and 50% reported Hy. Group B2 was completely asymptomatic. When assessing the ovarian reserve of the patients included in the pathological group, we found that patients in the A1 subgroup had Anti-Müllerian Hormone (AMH) values ranging from 1.9 to 4 ng/mL, with a mean value of 2.82 ng/mL (±0.7 ng/mL). In contrast, patients in the A2 subgroup had AMH values ranging from 0.01 to 0.5 ng/mL, with a mean value of 0.21 ng/mL (±0.15 ng/mL). A statistically significant difference was observed between the two groups, t(67) = 27.966, *p* < 0.05.

Considering the severe symptomatology associated with the A2 subgroup and the markedly reduced ovarian reserve, the patients opted for surgical intervention.

### 3.3. Uterine Size and Morphology

**Figure 2:**
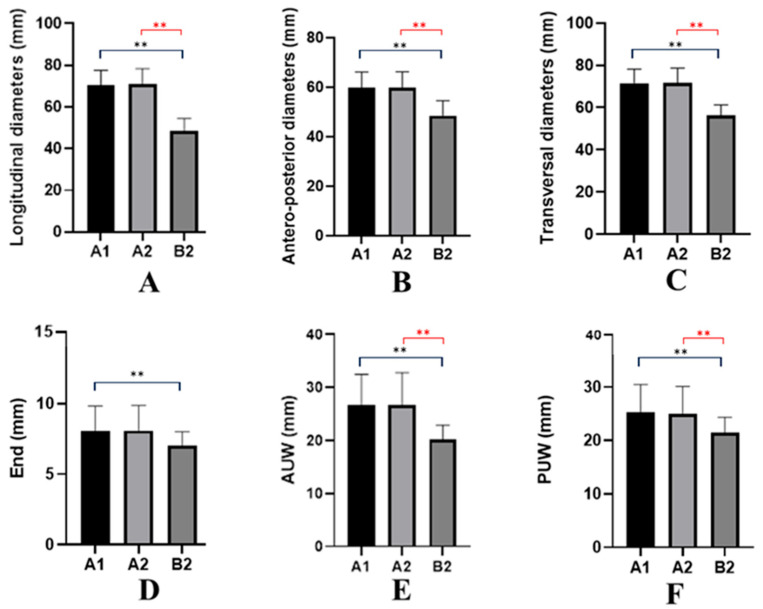
(**A**–**F**). Comparison of Uterine Dimensions Between Study Groups. Bars represent mean ± SEM. For all parameters, one-way ANOVA was applied, followed by Tukey’s post hoc comparisons. (**A**) Longitudinal diameter. Significant group effect (F(2,117) = 78.94, *p* < 0.0001). A1 and A2 did not differ, while both were significantly higher than B2 (*p* < 0.0001); (**B**) Antero-posterior diameter. Significant group effect (F(2,117) = 26.99, *p* < 0.0001). No difference between A1 and A2; both were significantly greater than B2 (*p* < 0.0001). (**C**) Transversal diameter. Significant group effect (F(2,117) = 45.50, *p* < 0.0001). A1 = A2; both significantly higher than B2 (*p* < 0.0001). (**D**) Endometrium thickness. Significant group effect (F(2,117) = 3.452, *p* = 0.0349). A1 = A2; A1 > B2 (*p* = 0.0361); A2 vs. B2 showed a non-significant trend (*p* = 0.0551). (**E**) AUW diameter. Significant group effect (F(2,117) = 11.34, *p* < 0.0001). A1 = A2; both significantly higher than B2 (*p* < 0.0001). (**F**) PUW diameter. Significant group effect (F(2,117) = 4.833, *p* = 0.0096). A1 = A2; both significantly higher than B2 (A1: *p* = 0.0092, A2: *p* = 0.0217). ns = not significant; ** in red = *p* < 0.01; A1: the group of patients with adenomyosis and primary infertility. A2: the group of patients with adenomyosis and secondary infertility. B2: the control group that included healthy patients whose imaging findings were used for comparative studies; AUW: Anterior Uterine Wall; PUW: Posterior Uterine Wall; End: Endometrium diameter.

Regarding the longitudinal diameter (L) of the uterine body, in Group “A1”, it ranged from 57 mm to 83 mm, with a mean value of 70.33 mm (±7.25 mm). In Group “A2”, it ranged from 57 mm to 83 mm, with a mean value of 70.95 mm (±7.47 mm). In Group “B2”, the longitudinal diameter ranged from 35 mm to 55 mm, with a mean value of 48.70 mm (±5.79 mm). A one-way ANOVA revealed significant differences in longitudinal diameter among the three groups (A1, A2, B2) (F(2,117) = 78.94, *p* < 0.0001, R^2^ = 0.5744). Tukey’s post hoc test showed no significant difference between A1 and A2 (*p* = 0.9054), indicating comparable mean values between the two subgroups of cohort A. In contrast, both A1 and A2 exhibited significantly higher longitudinal diameters compared with B2 (*p* < 0.0001 for both comparisons) (Figure 2A).

The antero-posterior diameter (AP) of the uterine body in Group “A1” ranged from 52 mm to 74 mm, with a mean value of 59.87 mm (±6.3 mm). In Group “A2”, it ranged from 52 mm to 74 mm, with a mean value of 59.83 mm (±6.38 mm), while in Group “B2”, it ranged from 38.6 mm to 59.1 mm, with a mean value of 48.53 mm (±6.06 mm). A one-way ANOVA revealed a significant group effect on the measured diameter (F(2,117) = 26.99, *p* < 0.0001, R^2^ = 0.3157). Tukey’s post hoc test showed no significant difference between A1 and A2 (mean difference 0.04, *p* = 0.9994), indicating that the two subgroups of cohort A exhibited virtually identical values. In contrast, both A1 and A2 showed significantly higher measurements compared with B2 (mean differences 11.34 and 11.30, respectively; *p* < 0.0001 for both comparisons) (Figure 2B).

The transverse diameter (T) of the uterine body in Group “A1” ranged from 56 mm to 83 mm, with a mean value of 71.53 mm (±6.70 mm). In Group “A2”, it ranged from 56 mm to 83 mm, with a mean value of 71.65 mm (±7.07 mm), while in Group “B2”, it ranged from 43 mm to 61 mm, with a mean value of 56.20 mm (±4.98 mm). A one-way ANOVA revealed a significant effect of group on the measured parameter (F(2,117) = 45.50, *p* < 0.0001, R^2^ = 0.4375). Tukey’s post hoc test showed no significant difference between A1 and A2 (mean difference −0.12, *p* = 0.9959), indicating that the two A subgroups exhibited nearly identical mean values. In contrast, both A1 and A2 displayed significantly higher measurements compared with B2 (mean differences 15.33 and 15.45, respectively; *p* < 0.0001 for both comparisons) (Figure 2C).

Regarding endometrial thickness (End), measured as a double layer during the proliferative phase, values in Group “A1” ranged from 5 mm to 12 mm, with a mean of 8.1 mm (±1.73 mm). In Group “A2”, values ranged from 5 mm to 12 mm, with a mean of 8.09 mm (±1.79 mm). In Group “B2”, values ranged from 5.6 mm to 8.3 mm, with a mean of 7.03 mm (±0.99 mm). A one-way ANOVA revealed a statistically significant difference among the three groups (F(2,117) = 3.452, *p* = 0.0349, R^2^ = 0.0557). Tukey’s post hoc test showed no significant difference between A1 and A2 (mean difference 0.0125, *p* = 0.9992). A1, however, presented a significantly higher mean value compared with B2 (mean difference 1.07, *p* = 0.0361), while the difference between A2 and B2 did not reach statistical significance (mean difference 1.06, *p* = 0.0551). These results indicate that only A1 differed significantly from B2, whereas A2 showed an intermediate, borderline pattern (Figure 2D).

The anterior uterine wall thickness (AUW) in Group “A1” ranged from 15 mm to 35 mm, with a mean value of 26.57 mm (±5.93 mm). In Group “A2”, thickness ranged from 15 mm to 35 mm, with a mean value of 26.71 mm (±6.07 mm), and in Group “B2”, it ranged from 17 mm to 25 mm, with a mean of 20.10 mm (±2.81 mm). A one-way ANOVA revealed a statistically significant effect of group on the measured variable (F(2,117) = 11.34, *p* < 0.0001, R^2^ = 0.1624). Tukey’s post hoc comparisons showed no significant difference between A1 and A2 (mean difference −0.146, *p* = 0.9911). In contrast, both A1 and A2 presented significantly higher values compared with B2 (A1 vs. B2: mean difference 6.467, *p* < 0.0001; A2 vs. B2: mean difference 6.613, *p* < 0.0001). These results indicate that the two A subgroups have similar values, while B2 is significantly lower than both (Figure 2E).

The posterior uterine wall thickness (PUW) in Group “A1” ranged from 15 mm to 35 mm, with a mean value of 25.20 mm (±5.29 mm); in Group “A2”, it ranged from 15 mm to 35 mm, with a mean value of 25.03 mm (±5.07 mm); in Group “B2”, it ranged from 16 mm to 26 mm, with a mean of 21.4 mm (±2.95 mm). A one-way ANOVA showed a statistically significant difference among the three groups (F(2,117) = 4.833, *p* = 0.0096, R^2^ = 0.0763). Tukey’s post hoc test revealed no significant difference between A1 and A2 (mean difference 0.175, *p* = 0.9833). In contrast, both A1 and A2 displayed significantly higher values compared with B2 (A1 vs. B2: mean difference 3.8, *p* = 0.0092; A2 vs. B2: mean difference 3.625, *p* = 0.0217). These findings indicate that the two A subgroups are comparable, whereas B2 shows consistently lower values (Figure 2F). Across all measured uterine parameters (L, AP and T diameters, End, AUW and PUW), the statistical analyses consistently showed no significant differences between A1 (A + PI) and A2 (A + SI). In contrast, both A1 and A2 presented significantly higher values compared with B2 (control group), indicating a uniform structural enlargement in the adenomyosis cohorts, while B2 consistently displayed lower measurements across all assessed variables.

### 3.4. Junctional Zone Alterations

**Figure 3:**
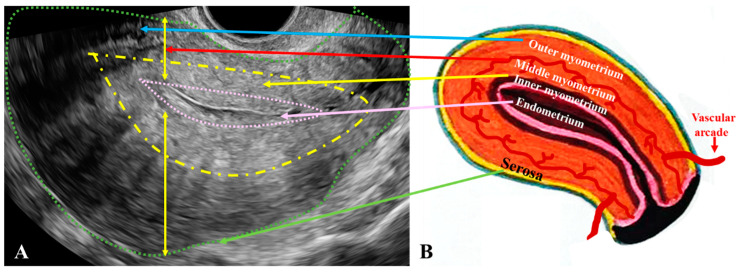
Normal and pathological aspects of the uterine wall layers. (**A**): Two-dimensional transvaginal ultrasound (2D-TVUS) image showing the endometrium (highlighted in pink) located internally. In adenomyosis (A), the junctional zone (JZ) shows marked thickening and a poorly defined external contour (yellow dashed line). The serosa, middle myometrium, and outer myometrium are delineated by the vascular arcade, with the outermost boundary (serosa) indicated by the green dashed line. (**B**): Schematic representation of normal uterine wall architecture, unaffected by adenomyosis. A: adenomyosis; 2D-TVUS: two-dimensional transvaginal ultrasound; JZ: endo-myometrial junctional zone (inner myometrium).

The muscular layer of the uterus is divided into three zones: the inner myometrium, also referred to as the JZ, located immediately beneath the endometrium and normally appears hypoechoic on ultrasound and it may appear thickened in the presence of A (Figure 3A); the middle myometrium is the portion situated between the JZ and the intramyometrial vascular arcade (Figure 3B); and the outer myometrium is the part between the vascular arcade and the serosa.

In patients included in Group “A” due to the presence of (A), we observed the appearance of hyperechogenic subendometrial lines or buds during 2D-TVUS and 3D-TVUS examinations, as well as the presence of myometrial cysts, reflecting endometrial tissue invading the myometrium.

**Figure 4:**
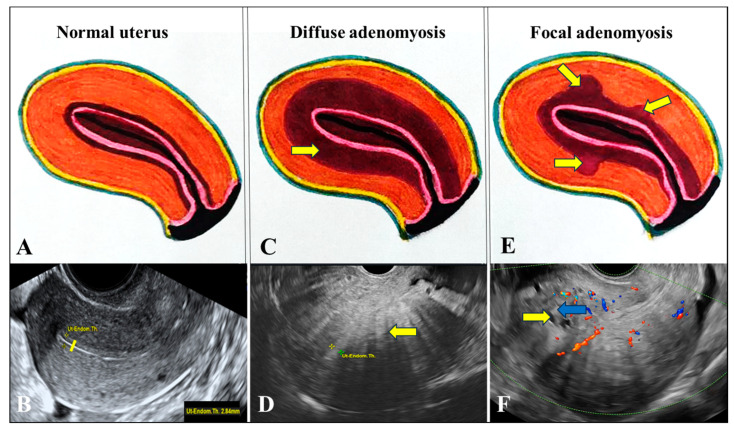
Schematic and ultrasound representations of the normal uterus (control) and an uterus affected by adenomyosis. (**A**): Schematic diagram of a normal uterus: the serosa is shown peripherally, the myometrium in orange, the junctional zone (JZ) in burgundy, and the endometrium in pink. (**B**): 2D transvaginal ultrasound (2D-TVUS) image of a normal anteverted uterus, displaying a thin trilaminar endometrium and a thin JZ (the yellow continuous line). (**C**): Schematic illustration of a uterus with diffuse adenomyosis, showing marked thickening of the JZ (brown). (**D**): 2D-TVUS image of diffuse adenomyosis, revealing hyperechogenic intramyometrial areas and the characteristic “Venetian blind” appearance (the yellow arrow). (**E**): Schematic diagram of a uterus with focal adenomyosis, highlighting localized JZ thickening (yellow arrows). (**F**): 2D-TVUS image of focal adenomyosis, showing focal JZ thickening, anechoic zones suggestive of intramyometrial endometrial cysts (the yellow arrow), and hyperechogenic areas consistent with smooth muscle cell (SMC) hyperplasia (the blue arrow). *JZ*: endo-myometrial junctional zone (inner myometrium); 2D-TVUS: two-dimensional transvaginal ultrasound; *SMC*: smooth muscle cells.

**Figure 5:**
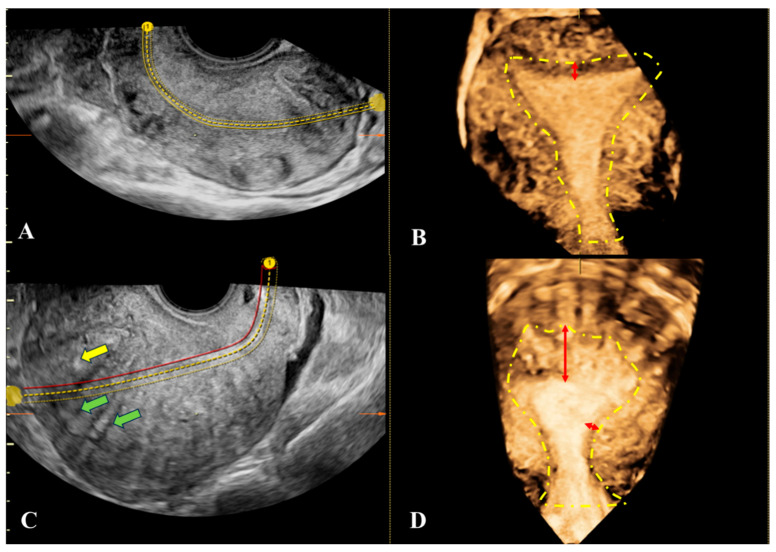
Ultrasound appearance of the junctional zone (JZ) in unaffected and adenomyosis-affected uterus. (**A**,**B**): 3D transvaginal ultrasound (3D-TVUS) images showing a clear view of the uterine cavity and a uniformly thin, hypoechoic JZ with normal and consistent dimensions in uteri without adenomyosis. (**C**,**D**): 3D-TVUS images showing the uterine cavity and a thickened JZ, especially in the right fundal region, compared to the rest of the JZ—suggestive of adenomyosis. The yellow dashed line marks the end of the JZ. The red arrow indicates the thickness of the JZ. JZ: endo-myometrial junctional zone (inner myometrium); 3D-TVUS: three-dimensional transvaginal ultrasound.

In the presence of A, the JZ appears irregular or interrupted. Differences in echogenicity between the uterine layers may be explained by variations in vascularization or tissue density. On 3D-TVUS, an interrupted JZ reflects the presence of invasive endometrial glands, in contrast to unaffected tissue, where the JZ appears continuous. A thickened and irregular JZ is indicative of SMC hyperplasia. On TVUS, SMC hyperplasia associated with A is thought to be responsible for JZ thickening and the presence of hyperechogenic islands (Figure 4A–F and Figure 5A–D).

Intramyometrial cystic formations were detected in 46% of patients in Group “A” (Figure 4F).

Hyperechogenic areas were visualized in all cases from Group “A” (Figure 4D).

Hypoechogenic striations, also described as a “Venetian blind appearance,” were observed in all Group “A” (Figure 4D).

**Figure 6:**
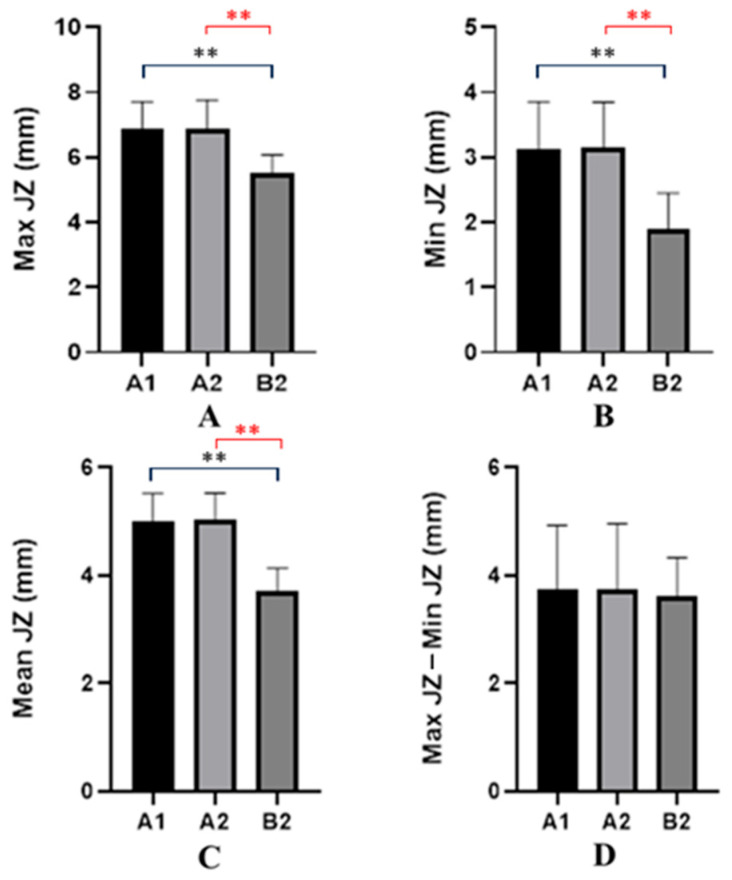
Variations in junctional zone (JZ) thickness (mm). (**A**) Maximum JZ thickness (JZmax). One-way ANOVA showed a significant group effect (F(2,117) = 23.32, *p* < 0.0001). Tukey’s test revealed no difference between A1 and A2, whereas both adenomyosis groups (A1 and A2) had significantly higher JZmax compared with controls (*p* < 0.0001). (**B**) Minimum JZ thickness (MinJZ). One-way ANOVA demonstrated a significant group effect (F(2,117) = 26.86, *p* < 0.0001). A1 and A2 did not differ, but both groups showed significantly higher MinJZ values compared with B2 (*p* < 0.0001). (**C**) Mean JZ thickness (Mean JZ). One-way ANOVA confirmed a significant group effect (F(2,117) = 57.11, *p* < 0.0001). No difference was observed between A1 and A2, while both showed significantly higher Mean JZ thickness than B2 (*p* < 0.0001). (**D**) Average JZ thickness (calculated as the mean of MaxJZ and MinJZ for each case). One-way ANOVA showed no significant differences among groups (F(2,117) = 0.1006, *p* = 0.9044), with Tukey’s post hoc analysis confirming the absence of pairwise differences. ns = not significant; ** in red= *p* < 0.0001. Max JZ: maximum junctional zone thickness; Min JZ: minimum junctional zone thickness; JZ: endo-myometrial junctional zone (inner myometrium); Max JZ−Min JZ: intra-case JZ thickness variation. A1: The group of patients with adenomyosis and primary infertility. A2: The group of patients with adenomyosis and secondary infertility. B2: The control group that included healthy patients whose imaging findings were used for comparative studies. Overall, the analysis of junctional zone parameters (Max JZ, Min JZ, and Mean JZ) showed that patients with adenomyosis—both those with primary infertility (A1) and secondary infertility (A2)—consistently exhibited thicker JZ measurements compared with healthy controls (B2), for all individual components (MaxJZ and MinJZ). Although the averaged JZ value (MeanJZ) did not differ significantly between groups, the separate evaluation of maximum and minimum JZ thickness clearly demonstrated that JZ structural alterations are characteristic of adenomyosis regardless of infertility type.

The maximum thickness of the JZ in Group “A1” patients ranged from 5.6 mm to 8.5 mm, with a mean value of 6.87 mm (±0.85 mm). In Group “A2” patients, maximum thickness ranged from 5.6 mm to 8.5 mm, with a mean value of 6.89 mm (±0.87 mm). In Group B1, JZ thickness ranged from 4.5 mm to 6 mm, with a mean of 5.51 mm (±0.56 mm). Analysis of the JZmax revealed significant differences among the three groups (A1, A2, B2), as shown by one-way ANOVA (F(2,117) = 23.32, *p* < 0.0001). Tukey’s post hoc test indicated no difference between A1 and A2, whereas both adenomyosis groups (A1 and A2) exhibited significantly increased JZmax compared with the control group B2 (*p* < 0.0001) (Figure 6A).

The minimum thickness of the JZ in Group “A1” patients ranged from 2 mm to 4 mm, with a mean value of 3.13 mm (±0.72 mm). In Group “A2”, patients ranged from 2 mm to 4 mm, with a mean value of 3.15 mm (±0.70 mm). In Group “B1”, JZ thickness ranged from 1 mm to 3 mm, with a mean of 1.9 mm (±0.55 mm). Statistical analysis of MinJZ thickness revealed a significant overall group effect (one-way ANOVA: F(2,117) = 26.86, *p* < 0.0001). Tukey’s post hoc test showed no significant difference between A1 and A2, whereas both groups with adenomyosis (A1 and A2) exhibited significantly higher MinJZ values compared with the control group B2 (*p* < 0.0001) (Figure 6B).

The mean value calculated between the maximum and minimum thicknesses of the JZ revealed that in the case of group “A1”there were average values between 4 mm and 5.5 mm, with an overall average of 5 mm (±0.51 mm); in the case of group “A2”, there were average values between 4 mm and 5.5 mm, with an overall average of 5.02 mm (±0.50 mm); and in the case of group “B2”, there were average values between 3 and 4.5, with an overall average of 3.71 (±0.43 mm). Analysis of the mean junctional zone thickness (Mean JZ) showed a significant overall difference between the three groups (A1 = adenomyosis with primary infertility, A2 = adenomyosis with secondary infertility, B2 = healthy controls), as demonstrated by one-way ANOVA (F(2,117) = 57.11, *p* < 0.0001). Post hoc Tukey testing revealed no significant difference between A1 and A2, whereas both groups, A1 and A2, exhibited significantly greater Mean JZ values compared with B2 (*p* < 0.0001 for both comparisons) (Figure 6C). Calculating the difference between the maximum thickness of JZ and its average thickness in group “A1”, we observed that there were values between 2 mm and 6.5 mm, with an overall average value of 3.73 mm (±1.19 mm); in group “A2”, we observed that there were values between 2 mm and 6.5 mm, with an overall average value of 3.74 mm (±1.22 mm); and in group B2, there were values between 2.5 mm and 5 mm, with an overall average value of 3.61 mm (± 0.72 mm). There was no significant difference in mean junctional zone (JZ) thickness among the three groups—A1 (adenomyosis with primary infertility), A2 (adenomyosis with secondary infertility), and B2 (healthy controls). One-way ANOVA showed no group effect (F(2,117) = 0.1006, *p* = 0.9044), and Tukey’s post hoc test confirmed that none of the pairwise comparisons reached statistical significance (A1 vs. A2: *p* = 0.9998; A1 vs. B2: *p* = 0.9076; A2 vs. B2: *p* = 0.912) (Figure 6D).

### 3.5. HyCoSy Findings

**Figure 7:**
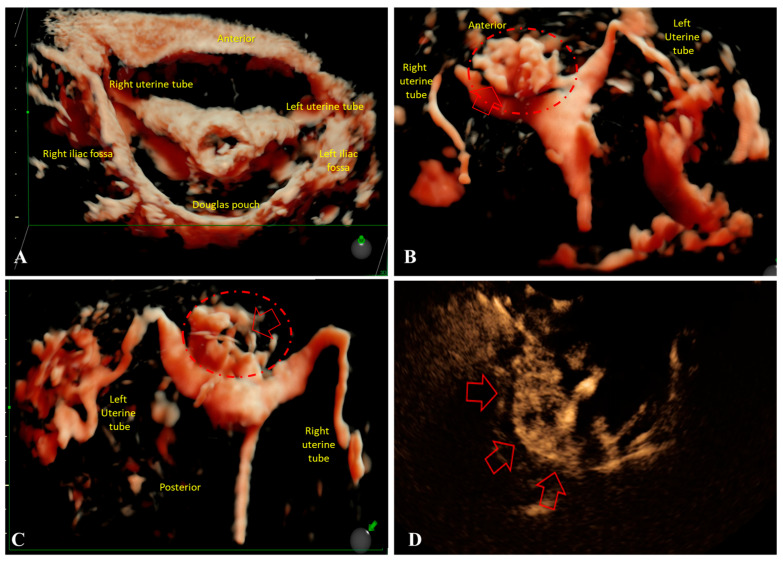
HyCoSy findings in patients with or without adenomyosis (A). (**A**): HyCoSy image from a patient with primary infertility (PI), showing normal contrast diffusion through the fallopian tubes and into the anterior uterine, posterior (retro-uterine), and bilateral peri-ovarian spaces. (**B**): Anterior HyCoSy view from a patient with PI and focal adenomyosis (moderate form), showing bilateral tubal patency and contrast accumulation in both peri-ovarian regions. The focal adenomyotic lesion, which does not reach the serosa, is outlined with a red dashed line (red arrows = contrast extravasation). (**C**): Posterior HyCoSy view from another patient with PI and focal adenomyosis (moderate form), again showing bilateral tubal patency and peri-ovarian contrast accumulation. The adenomyotic focus is outlined with a red dashed line (red arrows = contrast extravasation). (**D**): 2D-TVUS image showing intramyometrial diffusion of the contrast agent (red arrows = contrast extravasation). HyCoSy: contrast-enhanced Hysterosalpingo Contrast Sonography; A: adenomyosis; PI: primary infertility; 2D-TVUS: two-dimensional transvaginal ultrasound.

Given the presence of PI in group “A1” or SI in group “A2”, tubal patency was assessed using contrast-enhanced HyCoSy. Through this procedure, we assessed the extent of structural alteration of the myometrium and the invasiveness of A. This procedure was also performed in control group “B2”. In 36 patients from Group “A” (60% of Group “A1”) and 28 patients from Group “A2” (70% of Group A2), focal A of moderate form was observed, characterized by partial invasion of the myometrium (Figure 7A–D).

**Figure 8:**
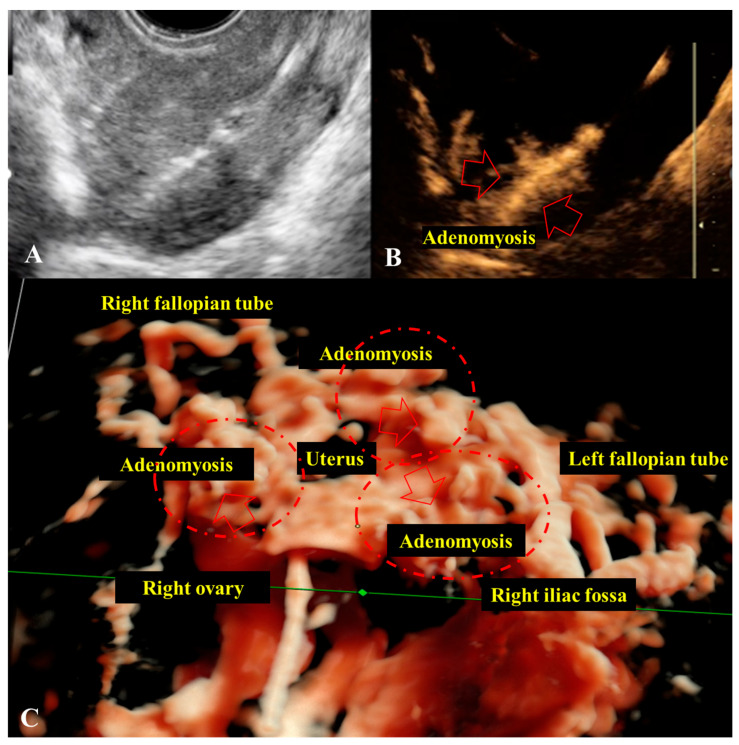
HyCoSy findings in severe cases of adenomyosis (A). (**A**): 2D transvaginal ultrasound (2D-TVUS) image from a patient with severe adenomyosis, captured during intrauterine instillation of contrast agent using a Foley catheter passed through the cervix. (**B**): Image acquired during contrast instillation, showing intramyometrial diffusion of the contrast agent extending to the serosal layer (red arrows = contrast extravasation). (**C**): Anterior HyCoSy view in a patient with primary infertility (PI) and severe adenomyosis, showing bilateral tubal patency and bilateral peri-ovarian contrast accumulation. Multifocal areas of deep, invasive adenomyosis reaching the serosal layer are highlighted by a red dashed line and red arrow (red arrows = contrast extravasation). A: adenomyosis; PI: primary infertility; 2D-TVUS: two-dimensional transvaginal ultrasound; HyCoSy: Hysterosalpingo Contrast Sonography.

In 12 patients from Group “A1” (20% of Group “A1”) and 12 patients from Group “A2” (30% of Group “A2”), multifocal zones of extensive myometrial invasion were detected, in some cases extending to the serosal layer (Figure 8A–C).

Additionally, among Group “A1” patients, 20 patients (33.33%) showed bilateral proximal tubal obstruction, 12 patients (20%) had unilateral distal tubal obstruction, 28 patients (46.66%) had bilateral tubal patency with contrast diffusion into the Douglas pouch, as well as the anterior uterine and peri-ovarian spaces. Among Group “A2” patients, 8 patients (20%) presented bilateral proximal tubal obstruction, 16 patients (40%) had unilateral distal tubal obstruction, 16 patients (40%) showed bilateral tubal passage and contrast diffusion into the Douglas pouch, anterior uterine, and peri-ovarian spaces.

### 3.6. Histopathology and Immunohistochemistry

**Figure 9:**
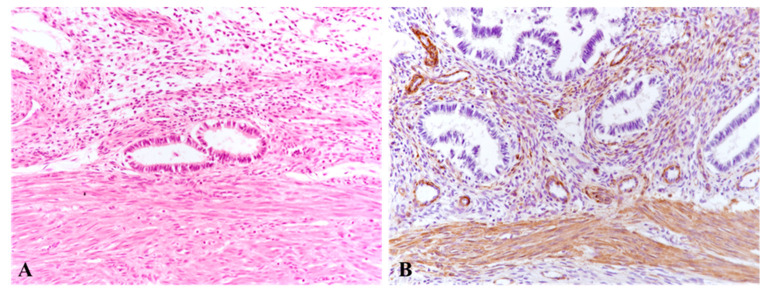
Normal microscopic aspects of the uterine wall (control cases, Group B1). (**A**): Normal architecture of the myometrial layers showing homogeneous smooth muscle cells (SMCs), regardless of the layer, oriented parallel to the endometrial glands in the junctional zone (JZ). No significant differences are observed in the distribution of loose connective tissue or intercellular collagen fibers. Hematoxylin and Eosin (HE) staining, ×200. (**B**): Uniform distribution of α-smooth muscle actin (α-SMA) within SMCs at the JZ, with no differences in immunohistochemical staining intensity between different myometrial layers. Immunohistochemistry with anti-α-SMA antibody, ×100SMCs: smooth muscle cells; JZ: endo-myometrial junctional zone; α-SMA: alpha-smooth muscle actin; HE: Hematoxylin and Eosin.

In the case of normal uterine structure, without the presence of A, the endometrial glands are properly localized within the uterine mucosa, without invading the myometrium or disrupting its architecture. The myometrium exhibits a homogeneous appearance, with no evident differences in tissue or cellular composition between its inner and outer layers. All three uterine layers are primarily composed of smooth muscle cells (SMCs) (Figure 9A), and no differences were observed between the layers regarding the α-SMA staining pattern of the SMCs or their count (Figure 9B). The SMCs in both the inner and outer myometrium displayed scant cytoplasm filled with myofilaments. No differences were identified in collagen distribution between the connective tissue of the inner myometrium (JZ) and the outer layer. In the inner myometrium, SMCs appeared densely interconnected within connective tissue, with a longitudinal orientation, running parallel to the endometrial glands. In contrast, in the outer myometrium, SMCs were more dispersed, with greater extracellular spacing (Figure 9A,B).

**Figure 10:**
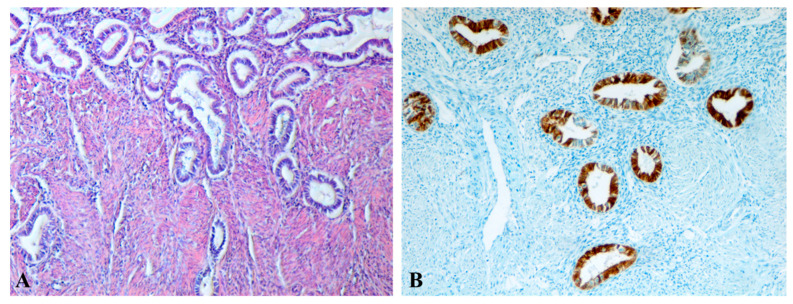
Microscopic aspects of the uterine wall affected by adenomyosis (cases from Group A2). (**A**,**B**): Presence of endometrial glands and periglandular stroma infiltrating toward the junctional zone (JZ), at a distance from the endometrial lining and deeply embedded within the myometrium. The JZ is disrupted by invasive endometrial glands penetrating the myometrial layers, disorganizing the architecture of smooth muscle cells (SMCs) and leading to the loss of their parallel alignment with the glands. SMCs appear hypertrophic and are separated by a prominent loose connective tissue matrix. (**A**): Classical Hematoxylin and Eosin (HE) staining, ×100. (**B**): Immunohistochemical staining with anti-CK7 antibody, ×100. A: adenomyosis; JZ: endo-myometrial junctional zone; SMCs: smooth muscle cells; HE: Hematoxylin and Eosin.

**Figure 11:**
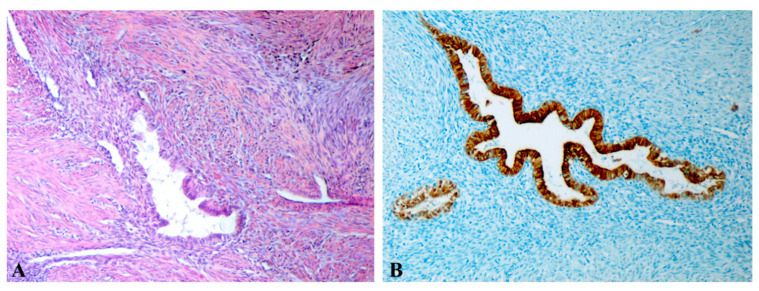
Microscopic aspects of the uterine wall affected by adenomyosis (Group A2 cases). (**A**): Endometrial glands and periglandular stroma are identified within the myometrial structure, leading to architectural disorganization. Smooth muscle cells (SMCs) appear hypertrophic and are separated by a prominent loose connective tissue matrix. Hematoxylin and Eosin (HE) staining, ×100. (**B**): Similar findings are observed: endometrial glands and periglandular stroma infiltrate the myometrium, disrupting its normal architecture. Immunohistochemical staining with anti-CK7 antibody, ×100. A: adenomyosis; SMCs: smooth muscle cells; HE: Hematoxylin and Eosin.

**Figure 12:**
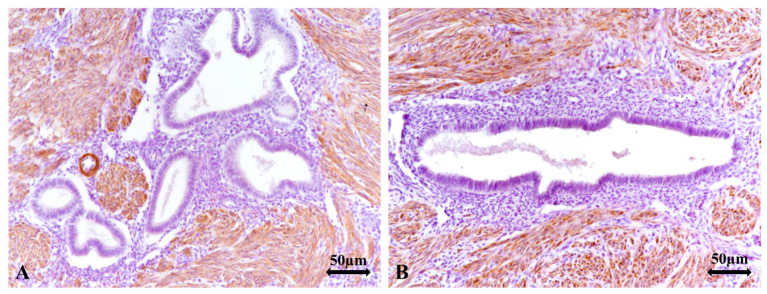
Microscopic aspects of the uterine wall affected by adenomyosis (A). (**A**): Disorganized inner myometrium with hypertrophic and sparsely distributed smooth muscle cells (SMCs), abundant extracellular matrix, numerous myofibroblasts, and signs of metaplasia—features indicative of chronic tissue injury and repair processes. Immunohistochemical staining with anti-α-SMA antibody, ×200. (**B**): Disorganized middle myometrium with hypertrophic SMCs and a dense extracellular matrix rich in myofibroblasts, showing strong α-SMA immunoreactivity. Immunohistochemical staining with anti-α-SMA antibody, ×200. A: adenomyosis; SMCs: smooth muscle cells; α-SMA: alpha-smooth muscle actin; HE: Hematoxylin and Eosin.

The histopathological diagnosis of A was established in surgically treated patients from the “A2” cohort by identifying endometrial glands and stroma located at a distance from the endometrium, embedded within the myometrial tissue. In case A, the inner myometrium appeared disrupted, with a loss of the parallel orientation between SMCs and the endometrial glands (Figure 10A,B). Basal endometrial glands and stroma infiltrated the myometrium, surrounded by collagen fibers, and could extend into the middle or even outer myometrium (Figure 11A,B). Additionally, at the level of the JZ, there were signs of cellular hypertrophy and metaplasia, with a reduction in the myocytes count, nuclear size, and cytoplasmic volume, along with dispersed SMCs separated by a well-developed loose connective tissue matrix. The increased intensity of α-SMA immunostaining in the uterine structure affected by A suggests the presence of myofibroblasts and metaplastic transformation as a response to chronic tissue injury and ongoing repair mechanisms (Figure 12A,B).

**Figure 13:**
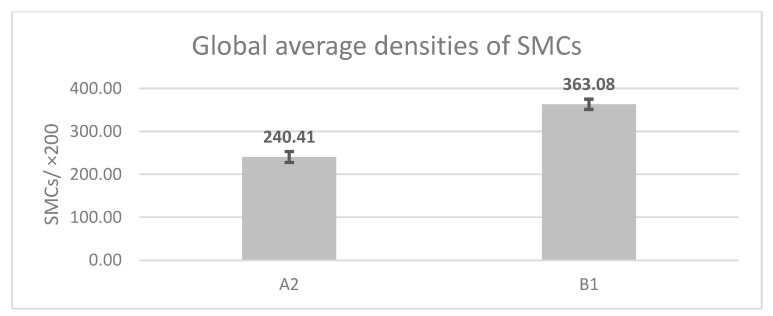
Global average densities of SMCs according to the analyzed group. A higher average cell density is observed in cases from control group B1 compared to the analyzed pathological group A2. Statistically significant differences were obtained in favor of control group B1: t(37) = −13.215, *p* < 0.05. A2: group of patients diagnosed with A and SI undergoing surgery; B1: control group comprising young women who died in road accidents; A: adenomyosis; SI: secondary infertility.

All immunohistochemical quantifications were performed blinded to group allocation. The mean cell density values for each case in group “A2” undergoing surgery ranged between 208.25 SMCs and 306 SMCs, with an overall mean value of 240.41 SMCs/×200 (±12.85 SMCs), while for the control group “B1”, SMC densities ranged between 322.25 SMCs and 405.75 SMCs, with an overall mean value of 363.08 SMCs (±11.94 SMCs). Analyzing the overall mean values of SMC density, we observed statistically significant differences in favor of group “B1”, t(37) = −13.215, *p* < 0.05 (Figure 13).

**Figure 14:**
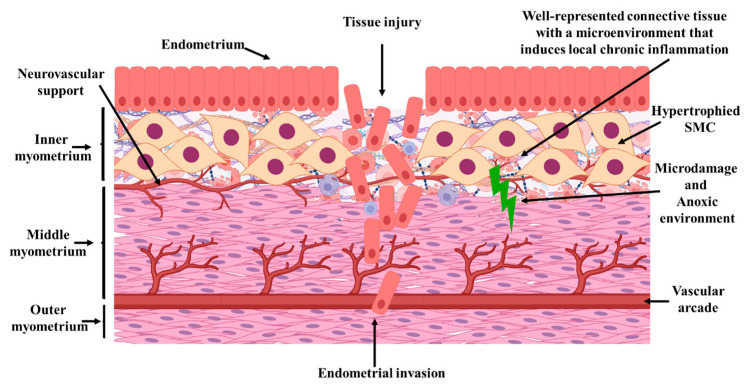
Illustration of Structural Changes in the Uterine Wall Due to Adenomyosis. The inner layer (endometrium) contains both normal endometrial glands and stroma, as well as ectopic glands extending beyond the junctional zone (JZ) into the outer myometrium, surpassing the vascular arcade. The JZ (inner myometrium) exhibits hypertrophied and sparsely distributed myocytes, abundant intercellular loose connective tissue rich in collagen fibers, and a pro-inflammatory microenvironment that sustains chronic local inflammation—factors that may negatively impact fertility. The middle myometrium contains invasive endometrial glands, which can extend as far as the outer myometrium. JZ: endo-myometrial junctional zone. Illustration created with BioRender. © Istrate-Ofițeru, A. (2025). https://BioRender.com/n6907uw.

**Figure 15:**
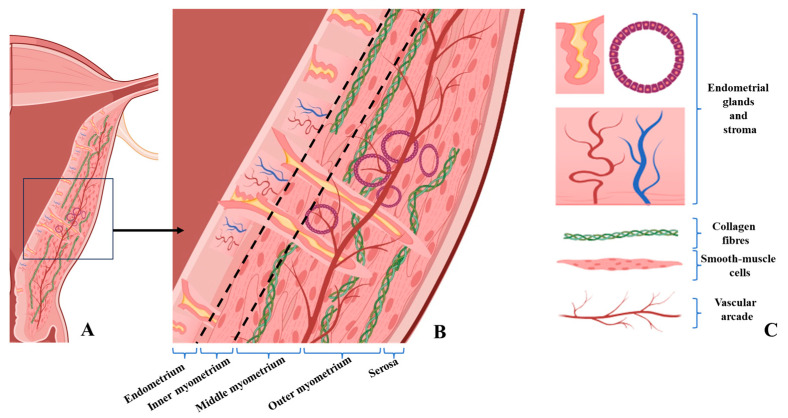
Schematic Illustration of Structural Alterations in the Uterine Wall Due to Adenomyosis. (**A**): Overview of the uterine wall illustrating structural alterations associated with adenomyosis. Illustration created with BioRender.com. © Istrate-Ofițeru, A. (2025). https://BioRender.com/6gmcpfp. (**B**,**C**): The inner layer (endometrium) contains both normal endometrial glands and stroma, as well as ectopic glands extending beyond the junctional zone (JZ) into the outer myometrium. The JZ (inner myometrium), delineated by black dashed lines, shows hypertrophic and sparsely distributed myocytes, along with a well-developed intercellular loose connective tissue matrix rich in collagen fibers. The middle myometrium displays invasive endometrial glands and dilated glands that may form intramural cystic structures. JZ: endo-myometrial junctional zone. Created in BioRender. Istrate-Ofiteru, A. (2025) https://BioRender.com/6gmcpfp.

Thus, a serrated JZ, invagination of endometrial glands, cellular hypertrophy or fibrosis in the inner and/or outer myometrium, and the presence of endometrial glands and stroma deeply embedded within the myometrium are considered hallmark histological features of A (Figure 14 and Figure 15A–C).

**Figure 16:**
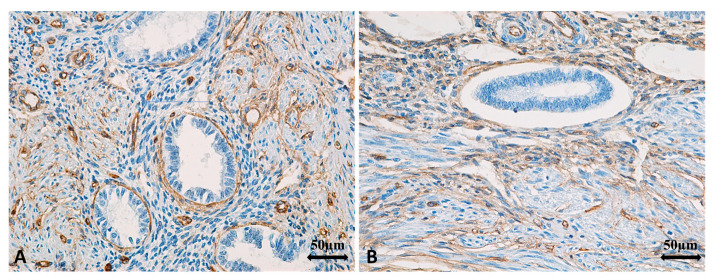
(**A**,**B**). Average CD34-positive global vascular densities according to the group of patients analyzed. (**A**): An increased count of vessels immunolabeled with anti-CD34 antibody at the endothelial level is observed in the pathological group of patients undergoing surgery—A2, ×200; (**B**): A lower density of vessels immunolabeled with anti-CD34 antibody at the endothelial level is observed in the control group, young victims of road accidents—group B1, ×200. CD: Cluster of differentiation. A2: group of patients diagnosed with A and SI undergoing surgery; B1: control group comprising young women who died in road accidents; A: adenomyosis; SI: secondary infertility.

**Figure 17:**
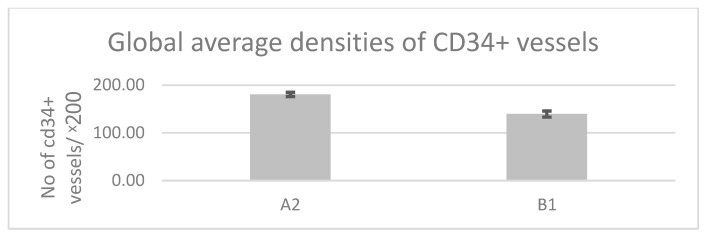
Global average densities of CD34+ vessels according to the analyzed group. A higher average vessels density is observed in cases from pathological group A2 compared to the analyzed control group B1. Statistically significant differences were obtained in favor of control group A2: t(28) = 25.603, *p* < 0.05. A2: group of patients diagnosed with A and SI undergoing surgery; B1: control group comprising young women who died in road accidents; A: adenomyosis; SI: secondary infertility.

Regarding the vascular density immunolabeled at the endothelial level with the anti-CD34 antibody, we observed that in the group of patients that underwent surgery—“A2,” the average vascular values ranged between 169.25 vessels/×200 and 184.75 vessels/×200, with an overall average value of 180.38 vessels/×200 (±4.36 vessels/×200), while in the control group, which included road accident victims, the average vascular density values ranged between 130.75 vessels/×200 and 144.75 vessels/×200, with an overall average value of 139.33 vessels/×200 (±6.47 vessels/×200). We observed a statistically significant difference in favor of group “A2” (Figure 16A,B and Figure 17), t(28) = 25.603, *p* < 0.05.

## 4. Discussion

Our study provides new insights into the morphological and structural changes associated with A in women with PI and SI, emphasizing the importance of JZ alterations in diagnostic imaging and histopathology. This study shows that, in patients with A, there is a significantly greater thickening of the JZ area and a higher percentage of alterations compared to patients without A, and these JZ alterations observed by three-dimensional ultrasound are associated with A [16].

We observed that patients with A (Group “A”) were significantly older compared to those in control group (Group “B”). This finding is consistent with the epidemiological profile described in previous studies, where A is more frequently diagnosed in women aged >40 years, often in multiparous women or those with a history of uterine surgery [2,3,4]. In line with Abu Hashim et al. [6], who reported a high prevalence of A in infertile populations, our results support the hypothesis that the disease may differentially affect fertility across age groups. JZ alterations in women with A represent an important factor contributing to infertility [43,44].

Our study employed modern imaging techniques to evaluate women with infertility and included in the analysis those patients who exhibited sonographic changes characteristic of this pathology. The main focus was placed on the ultrasonographic and histopathological assessment of the JZ.

Direct sonographic signs of A—including hyperechogenic sub endometrial lines, myometrial cysts, and hypoechogenic striations—were consistently observed in our cohorts. These findings align with those of Exacoustos et al. [16] and Van den Bosch et al. [22], who emphasized the diagnostic weight of direct sonographic features over indirect signs. In our study, intramyometrial cysts were more prevalent in A1 patients, while hypoechogenic striations were frequent in both groups (A1, A2). This may suggest that specific imaging features could predominate depending on age and reproductive history, as also noted by Tellum et al. [24,30].

All uterine diameters were significantly larger in group A patients with A compared to group B patients. Enlargement of the uterus in A has been consistently described in the literature [1,45], although the correlation between uterine size and infertility remains debated. Our findings suggest that increased uterine size may be more pronounced in women diagnosed with A.

Although uterine dimensions were significantly larger in women with adenomyosis compared to controls, this finding must be interpreted with caution given the age difference between the groups. Uterine size naturally increases with age, cumulative estrogen exposure, and parity, independently of A. Therefore, part of the observed enlargement in Group (A) may reflect age-related physiological changes rather than A alone. This age-related effect has been noted in previous studies evaluating uterine morphology across reproductive age ranges and represents a potential confounding factor in our analysis.

One of the most relevant findings of our study is the significantly greater maximum and mean JZ thickness in both A1 and A2 group compared to group B2. These results are consistent with the literature describing JZ thickening and irregularity as hallmark sonographic features of A [12,15,16,21,22]. Tellum et al. [24] and Rasmussen et al. [19] highlighted that JZ > 5 mm is strongly associated with A, while Harmsen et al. [15] emphasized the diagnostic relevance of irregular or interrupted JZ in the updated MUSA consensus.

The specific parameters used to assess the junctional zone—such as JZmax, JZmin, and the JZmax − JZmin difference—have important clinical relevance. JZmax reflects the degree of focal thickening and is associated with local invasion of endometrial tissue, while JZmin provides a reference for baseline myometrial thickness. The difference between JZmax and JZmin is considered a marker of JZ irregularity and structural distortion, both of which have been linked to impaired uterine peristalsis, altered sperm transport, and disrupted endometrial–myometrial communication. Several studies have shown that increased JZ thickness (>5 mm) or marked asymmetry predicts reduced implantation rates, poorer IVF outcomes, and greater disease severity. Therefore, the detailed evaluation of these parameters provides clinically meaningful information about the functional integrity of the JZ and its potential contribution to infertility.

Tocci et al. suggested that the “endometrial–subendometrial myometrium unit (JZ) disruption disorder” should be regarded as a distinct condition from adenomyosis, mainly characterized by pathological changes or abnormal thickening of the junctional zone (JZ) [46]. According to other studies, the proliferation and excessive growth of smooth muscle tissue within the JZ may represent early stages that precede the expansion of endometrial cells and the development of adenomyosis [46,47,48].

In our study, we found that both patients with PI (Group A1) and those with SI (Group A2) had a maximal JZ thickness greater than 5 mm, findings consistently associated with A. Additionally, in the A group, the average thickness calculated from the maximum and minimum JZ measurements also exceeded 5 mm. These observations suggest that A can be present even in younger, nulliparous patients, but appears more frequently in women with a history of at least one prior pregnancy reinforcing the role of JZ thickening in patients with SI, suggesting that repeated pregnancies and uterine trauma may accentuate these changes, as also proposed by Kishi et al. [38].

Although our findings demonstrate significantly greater JZ thickness in women with adenomyosis, these differences must be interpreted with caution. JZ morphology is known to change with age, increasing naturally in thickness and irregularity across the reproductive years. Therefore, part of the JZ alterations observed in our cohort—particularly in the older A2 subgroup—may reflect age-related physiological remodeling rather than adenomyosis-specific changes alone. This age-related variability represents an important confounding factor and should be considered when comparing JZ parameters between groups.

The clinical utility of JZ evaluation is increasingly recognized in reproductive medicine. Beyond its diagnostic value, accumulating evidence suggests that JZ thickness and structural irregularity may also serve as predictors of reproductive outcomes and treatment response. Several studies have reported that an increased or heterogeneous JZ is associated with impaired uterine peristalsis, altered sperm transport, reduced implantation rates, and poorer ART outcomes, indicating that JZ morphology could help identify women at higher risk of subfertility or implantation failure [7,48,49]. Furthermore, emerging data show that medical therapies such as dienogest or GnRH analogs may reduce JZ thickness and improve uterine function, suggesting a potential link between JZ remodeling and therapeutic benefit [50]. In this context, our findings reinforce the concept that JZ assessment—particularly maximal thickness exceeding 5 mm—may have prognostic relevance in clinical decision-making, providing an opportunity for individualized fertility counseling and tailored treatment strategies.

Although contrast-enhanced HyCoSy is primarily designed to evaluate tubal patency, our findings suggest that it can also provide complementary information on uterine morphology in A. In our cohort, HyCoSy revealed both focal and multifocal invasion patterns of the myometrium, which correlated with structural changes detected by 2D/3D-TVUS with similar distribution between A1 and A2 groups. Previous studies have emphasized the role of three-dimensional sonography in assessing JZ and its correlation with histopathology [16], as well as the importance of standardized sonographic criteria such as those proposed by the MUSA group [15,22]. In this context, HyCoSy may serve as an ancillary tool, offering simultaneous assessment of tubal status and uterine structure, particularly relevant in the infertility work-up. Although HyCoSy revealed focal and multifocal patterns of intramyometrial contrast leakage suggestive of adenomyotic invasion, we did not perform quantitative measurements of invasion depth. At present, standardized criteria for quantifying the depth of myometrial infiltration on contrast-enhanced HyCoSy are lacking, and the technique is predominantly used qualitatively to assess the presence and distribution of leakage rather than precise millimetric penetration. In our study, HyCoSy findings were therefore interpreted in correlation with 2D/3D-TVUS morphology rather than as standalone quantitative metrics. We acknowledge this as a limitation, and future methodological developments—such as volumetric contrast analysis or automated tracking of contrast dispersion—may enable more robust quantification of invasion depth.

While HyCoSy provided valuable qualitative information on intramyometrial contrast leakage and its distribution, the diagnostic implications of these findings must be interpreted cautiously. HyCoSy is not validated as a diagnostic tool for A, and in our study, it was not systematically compared with a gold standard such as MRI or histopathology for all participants. Therefore, the observed invasion patterns should be considered complementary observations rather than definitive markers of A. Further studies incorporating direct validation are needed before HyCoSy can be reliably integrated into diagnostic algorithms for A.

Importantly, tubal obstruction—either proximal or distal—was more frequently observed in Group “A1”. This suggests that infertility in younger women may be multifactorial, combining A-related uterine changes with compromised tubal patency. While HyCoSy is not yet included among the standardized imaging criteria for A [24,30], its combined use with 3D-TVUS could increase diagnostic accuracy and help identify patients at higher risk of impaired fertility.

Diagnosing A through high-resolution ultrasound and contrast-enhanced HyCoSy has important implications for infertility management. The early identification of JZ thickening, irregularity, or intramyometrial invasion patterns can guide individualized therapeutic strategies aimed at optimizing reproductive outcomes. Several studies have shown that medical pretreatments—such as GnRH agonists or dienogest—may improve implantation conditions and clinical pregnancy rates in women with adeno-myosis undergoing ART [48,49]. In selected cases, uterine-sparing interventions, including adenomyomectomy, may further enhance fertility potential, particularly in younger patients with focal disease [50]. Moreover, identifying adenomyosis on ultrasound can influence IVF planning by guiding decisions on modified stimulation protocols, freeze-all strategies, or delaying embryo transfer to allow adequate endometrial recovery [51]. Taken together, our findings suggest that three-dimensional transvaginal ultra-sound, when complemented by qualitative information from HyCoSy, may offer useful insights into uterine structural alterations associated with adenomyosis in the infertility work-up. However, given the study’s retrospective design, the limited number of histologically confirmed cases, and the absence of systematic MRI validation, this combined approach should be regarded as a promising exploratory tool rather than a fully established diagnostic method.

Our findings also align with recent efforts to standardize the sonographic evaluation of A. The 2019 ultrasound-based classification proposed by Van den Bosch et al. [12] provides a structured framework for describing lesion type, extent, and junctional zone abnormalities, reinforcing the importance of consistent terminology in clinical and research settings. Although variability in JZ measurements on 3D-TVUS is acknowledged in the literature, especially regarding reconstruction angle and operator technique, the strong concordance between imaging and histology in our cohort supports the reliability of advanced ultrasound when performed using standardized criteria. These developments highlight the growing movement toward harmonized reporting of A and underscore the need for continued refinement of imaging-based classification systems.

Although this study was not primarily designed to evaluate diagnostic accuracy, the subset of 40 patients who underwent hysterectomy allowed us to make observations regarding the diagnostic performance of ultrasound. All 40 women showed 2D/3D-TVUS features highly suggestive of A—including JZ thickening >5 mm, myometrial cysts, hyperechoic islands, and irregular or disrupted JZ contours—and all were subsequently confirmed to have A on histopathological analysis. This complete concordance indicates a high positive predictive value of 2D/3D ultrasound for detecting moderate-to-severe A in this selected cohort. These findings are consistent with previous studies demonstrating strong agreement between advanced transvaginal ultrasound features and histopathology, particularly in patients with pronounced morphological changes and when examinations are performed by experienced operators [16,52]. While the retrospective design and selection bias limit formal sensitivity or specificity estimation, this observation reinforces the clinical reliability of ultrasound in identifying A in symptomatic infertile women.

In the A2 subgroup, hysterectomy was not performed for infertility itself but was indicated due to severe A-related symptoms. Most patients experienced a combination of debilitating Dy, Men, and, in several cases, Met or Hy, all contributing to significant impairment of quality of life. These symptoms persisted despite medical management. Moreover, patients in A2 exhibited markedly reduced ovarian reserve (mean AMH 0.21 ± 0.15 ng/mL), which further limited reproductive potential and influenced the decision toward definitive surgical treatment. Thus, hysterectomy in this group primarily reflected symptom severity rather than infertility management.

In A, the uterine JZ shows thickening and discontinuities caused by the invasion of endometrial tissue into the myometrium. Normally, the JZ presents a smooth transition between the endometrium and the myometrium, but in A, this boundary becomes irregular and blurred, containing endometrial glands and stroma within the muscle wall. This process results in typical microscopic changes, such as small cystic formations, hemorrhage, and a thickened, uneven appearance visible on imaging [53].

Histology confirmed the presence of ectopic endometrial glands and stroma within the myometrium, associated with SMC disorganization, hypertrophy, and metaplasia.

While two studies reported no difference in α-SMA expression between uteri with and without A [13,38], our findings demonstrated a reduction in SMCs density, resulting from cell hypertrophy and structural disorganization due to the invasion of endometrial glands, together with an increase in CD34+ vascular density, indicating intensified angiogenesis and ongoing tissue remodeling processes described by Ibrahim et al. [36] and Mehasseb et al. [35]. Collagen remodeling and disruption of the parallel orientation between SMCs and endometrial glands further support the hypothesis that A involves altered myometrial contractility and extracellular matrix remodeling [31,38,39]. These changes may explain impaired uterine peristalsis and suboptimal conditions for implantation, as also discussed by Tamura et al. [11] and Vercellini et al. [7].

The schematic diagrams were designed to synthesize and visually illustrate the key histopathological findings observed in our study. The depicted features—including glandular invasion beyond the junctional zone, disruption of smooth muscle cell orientation, expansion of loose connective tissue rich in collagen fibers, and the increased vascular network—directly reflect the microscopic changes documented on HE and immunohistochemical staining (α-SMA and CD34). These diagrams therefore serve as an integrative visual summary that complements the histological images by contextualizing how the structural alterations detected microscopically manifest within the broader architecture of the uterine wall.

Our results underline the clinical significance of JZ assessment in A-related infertility. The finding that JZ thickening is more pronounced in group A2 of patients suggests a progressive nature of the disease, with cumulative effects over time and parity. This supports the use of JZ thickness as a prognostic marker in reproductive outcomes, consistent with recent reviews [8,9]. Furthermore, the comparable diagnostic performance of 3D-TVUS and MRI reported by Alcázar et al. [25] highlights the practicality of ultrasound as a first-line tool in infertility work-up.

The present study has several limitations. The sample size was relatively small, and the retrospective design may introduce selection bias. Histological confirmation of A was available only for hysterectomy specimens, using standard Hematoxylin–Eosin staining as well as immunohistochemical staining with the anti-CK7 antibody, which highlighted the presence of endometrial glands within the myometrial structure (Group A2), limiting direct histological–imaging correlation in Group A2. Additionally, the coexistence of tubal obstruction and A complicates the attribution of infertility to A alone. These challenges have been repeatedly emphasized in the literature [5,7,11,30].

A major limitation of this study is the statistically significant age disparity between the adenomyosis group and the control group. Age is known to influence several reproductive and uterine parameters, including junctional zone thickness, uterine volume, ovarian reserve, and the prevalence and severity of symptoms. Therefore, part of the structural and functional differences observed between groups—such as increased JZ thickness, uterine enlargement, and reduced AMH levels—may be attributable to age-related physiological changes rather than adenomyosis alone. This represents a significant confounding factor, and our findings must be interpreted with caution. Future studies with age-matched control groups are needed to identify how adenomyosis may influence these parameters. Interpretation of the literature concerning the JZ must be approached with caution, given the variability between imaging modalities (such as TVUS) and the absence of a standardized definition. While conventional histology does not reveal the functional characteristics of the JZ, imaging studies increasingly suggest a significant functional role of this structure in uterine pathologies, including A.

Future research should focus on validating our findings in larger, prospective, and preferably multicenter cohorts to reduce the impact of sample size and selection bias. Standardization of diagnostic criteria, particularly through the integration of updated MUSA definitions, is needed to improve the reproducibility of imaging assessments. The correlation between ultrasound phenotypes of A and reproductive outcomes should be further explored, ideally by incorporating detailed information on embryo implantation and assisted reproductive technology (ART) success rates.

There is a pressing need to develop a unified definition of A that includes clear criteria for both histopathological and imaging evaluation. Translational studies combining imaging findings with molecular and immunohistochemical profiling could help refine phenotype-based classifications of A and clarify the underlying mechanisms linking JZ alterations to infertility.

Moreover, the integration of artificial intelligence and machine learning algorithms into ultrasound image analysis has the potential to enhance diagnostic accuracy and reduce inter-observer variability.

Furthermore, longitudinal studies starting from adolescence are warranted to monitor progressive changes in the JZ and their potential evolution toward A.

## 5. Conclusions

Our study emphasizes the importance of JZ alterations in the pathophysiology of A-associated infertility.

Direct ultrasound signs of A, including hyperechoic sub endometrial lines, myometrial cysts, hypoechoic striations, and JZ irregularity, were consistently observed, with distinct patterns across the two infertility groups. HyCoSy revealed partial or multifocal myometrial invasion in most cases, as well as tubal obstruction in a subset of patients, underlining the multifactorial nature of infertility in A.

Histopathological and immunohistochemical findings confirmed endometrial gland invasion, SMC disorganization, increased CD34+ vessels density in JZ, decrease in SMCs in JZ, collagen remodeling, and increased α-SMC expression, supporting the role of chronic microtrauma and repair mechanisms in disease progression.

While our observations suggest that three-dimensional transvaginal ultrasound, complemented by qualitative information from HyCoSy, may offer valuable insights into uterine structure in the context of A, these conclusions must be interpreted with caution. The retrospective design, the limited number of histologically confirmed cases, and the lack of systematic comparison with a gold-standard modality (such as MRI) restrict the extent to which diagnostic utility can be fully established. Larger, prospective, and preferably age-matched studies are needed to validate these findings, refine diagnostic criteria, and strengthen correlations with reproductive outcomes.

## Figures and Tables

**Table 1:** Antibodies used in the immunohistochemical study.

Antibody	Producer	Species, Clone	Antigen Retrieval	Dilution	Target Label
Anti-CK7	Dako	Mouse, OV-TL 12/30	Citrate, pH 6.0	1:50	Endometrial epithelium
Anti-α-SMA	Dako	Mouse, 1A4	Citrate, pH 6.0	1:100	Smooth muscle actin
Anti-CD34	Dako	Mouse, QBEnd/10	Citrate, pH 6.0	1:50	Neoformed blood vessels

Abbreviations: α-SMA, alpha-smooth muscle actin; CK7, cytokeratin 7; CD 34: Cluster of differentiation 34.

## Data Availability

All data presented here are available from the authors upon reasonable request.

## Abbreviations

The following abbreviations are used in this manuscript:

2D-TVUS	two-dimensional transvaginal sonography three-dimensional transvaginal sonography
3D-TVUS	three-dimensional transvaginal sonography two-dimensional transvaginal sonography
A	Adenomyosis
AMH	Anti-Müllerian Hormone
ART	Assisted Reproductive Technologies
CD34	Cluster of Differentiation 34
CK7	Cytokeratin 7
Dy	Dysmenorrhea
HE/H&E	Hematoxylin–Eosin
Hy	Hypertension
HyCoSy	Hysterosalpingo-contrast sonography
IVF	In Vitro Fertilization
JZ	junctional zone
JZmax	the maximum thickness of the junctional zone
JZmin.	the minimum thickness of the junctional zone
Men	Menorrhagia
Met	Metrorrhagia
MRI	Magnetic Resonance Imaging
MUSA	Morphological Uterus Sonographic Assessment
PI	Primary infertility
SI	Secondary Infertility
SMC	Smooth muscle cells
TLA	Three letters acronyms
TVUS	Transvaginal Ultrasound
α-SMA	Alpha-Smooth Muscle Actin
